# Non-Destructive Measurement of Three-Dimensional Plants Based on Point Cloud

**DOI:** 10.3390/plants9050571

**Published:** 2020-04-29

**Authors:** Yawei Wang, Yifei Chen

**Affiliations:** 1 College of Information and Electrical Engineering, China Agricultural University, Qinghuadonglu No. 17, HaiDian District, Beijing 100083, China; yaweiwanghb@163.com; 2 Engineering Practice Innovation Center, China Agricultural University, Qinghuadonglu No. 17, HaiDian District, Beijing 100083, China

**Keywords:** point cloud processing, plant model, leaf feature point, 3D reconstruction, phenotype measurement

## Abstract

In agriculture, information about the spatial distribution of plant growth is valuable for applications. Quantitative study of the characteristics of plants plays an important role in the plants’ growth and development research, and non-destructive measurement of the height of plants based on machine vision technology is one of the difficulties. We propose a methodology for three-dimensional reconstruction under growing plants by Kinect v2.0 and explored the measure growth parameters based on three-dimensional (3D) point cloud in this paper. The strategy includes three steps—firstly, preprocessing 3D point cloud data, completing the 3D plant registration through point cloud outlier filtering and surface smooth method; secondly, using the locally convex connected patches method to segment the leaves and stem from the plant model; extracting the feature boundary points from the leaf point cloud, and using the contour extraction algorithm to get the feature boundary lines; finally, calculating the length, width of the leaf by Euclidean distance, and the area of the leaf by surface integral method, measuring the height of plant using the vertical distance technology. The results show that the automatic extraction scheme of plant information is effective and the measurement accuracy meets the need of measurement standard. The established 3D plant model is the key to study the whole plant information, which reduces the inaccuracy of occlusion to the description of leaf shape and conducive to the study of the real plant growth status.

## 1. Introduction

With the development of protected vegetable production, vegetable seedlings are increasingly recognized by producers, which has become an important pillar of vegetable industry development. In agriculture, information about the spatial distribution of crop growth is valuable for applications such as biomass and yield estimation or increasing field work efficiency in terms of fertilizing, applying pesticides and irrigation [1,2]. Many researchers have carried out experiments on plant growth detection based on computer vision technology, mainly covering three aspects: the detection of external growth parameters, fruit maturity, and the detection of nutritional components [3].

Plant height is an important phenotypic morphology parameters that can be used not only as an indicator of overall plant growth vigor, but a parameter to estimate traits [4,5]. The key technology to obtain plant height is to present the highest and the lowest points on the image (or point cloud), and obtain the height by distance formula [6,7,8]. Using the Euclidean distance [9,10] to present the plant height is commonly used on 2D images, extracting the plant skeleton on the region of interest (ROI) and detecting the lowest and highest points of the skeleton. The accuracy of pixel extraction in the interest domain of two-dimensional images is an influencing factor of plant height measurement accuracy. Image processing is used to segment the skeleton, such as color space calibration, or morphological method to present the skeleton. Then the height of the plant can be obtained according to the marker points or mapping the color space mapped to the depth space. Calcuation the plant height by vertical distance [11,12,13,14,15] is a method on 3D point clouds, calculating the difference of lowest and highest point of the height-axis on the ROI. In the study of 3D model, some researchers computed the mesh distance [16,17] of every rasterized plant point to the ground. In the process of three-dimensional plant height, the focus is the accuracy of modeling, especially in the field experiment, the spacing between plants, noise points of the soil and weeds are the key factors affecting the measurement accuracy. When measuring the plant’s height indoors, the sensors are often placed horizontally with the object, so the height’s coordinate system is the *y*-axis; when measuring the plant’s height outdoors, the sensors are placed vertically to the ground, so the height coordinate system is the *z*-axis.

Leaf is an important organ of vegetation, it determines the primary production of photosynthesis, plant evaporation and characterization of plant growth [18]. In a study of automatic measurements for plant leaves, the method of segmenting leaf based on two-dimensional images processing is widely used, for example, tobacco [19], leafy vegetables [20], pest-damaged leaf [21]. Further, the stereo vision method can also be used for leaf segmentation and measurement [18,22,23]. With the development of three-dimensional vision, more researchers segment the main organs of the plant after reconstruction the whole plant, extract the leaves, and study morphological features of leaves. For the point cloud organ segmentation method, the currently commonly used methods are segmentation of the whole plant by region growing algorithm [24,25,26], segmentation of the plant by locally convex connected patches (LCCP) segmentation [27,28], segmentation of the plant by Histogram clustering algorithm [29], segmentation starting finding the stem in a 3D point cloud and clustering the leaves by geometrical constraint [30,31].

Some researchers have also developed measurement systems to reconstruct 3D point cloud for plant of growth information [32,33,34,35,36], for example, Andújar et al. [37] explored the possibilities of using Kinect Fusion algorithms to reconstruct 3D point clouds of weed-infested maize crops under real field conditions. Yamamoto et al. [38] measured growth information of strawberry in greenhouse through the color and depth images by Kinect. Li et al. [39] developed a technique system for the measurement, reconstruction, and trait extraction of rice canopy architectures. The method of using Kinect and turntable to perform phenotypic information for indoor plants is necessary to rotate the turntable multiple times interval at a fixed angle [40,41,42,43], and then use the point cloud registering algorithm for 3D reconstruction.

**Figure 1:**
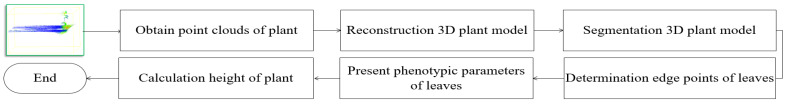
Flowchart of this paper for obtaining a plant’s morphological parameters.

In this paper, a new method of the reconstruction point cloud method for the greenhouse plant’s morphological parameters based on Kinect v2.0 and turntable is explored. The processed 3D plant model contains spatial information, and then the key growth parameters, leaf’s length, width, surface area and plant height are extracted. The specific flow of this article is shown in Figure 1, firstly, obtaining the point clouds of the plant by rotating the turntable 8 times intervals, and reconstruction of the 3D plant model by the registering method; secondly, segmenting the leaves of the 3D model and determining the edge points of leaves, presenting the phenotypic parameters of leaves; finally, calculating the plant’s height by vertical distance.

## 2. Preprocessing of a Three-Dimensional Plant Model

**Figure 2:**
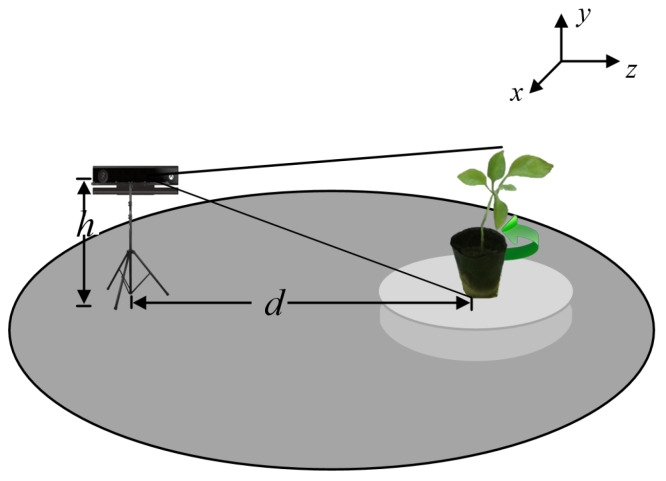
Measurement system of plant phenotypic parameters.

In this experiment, a Kinect camera and an electric turntable were used to build a 3D plant model according to the following instructions. Fix the camera position and put the plant on the turntable, the height *h* of Kinect is set to 0.7 m, and the distance *d* between Kinect and plant is 1.0 m. Figure 2 shows the experiment devices which are placed at fixed positions, and marks the world coordinate system of the point cloud.

**Figure 3:**
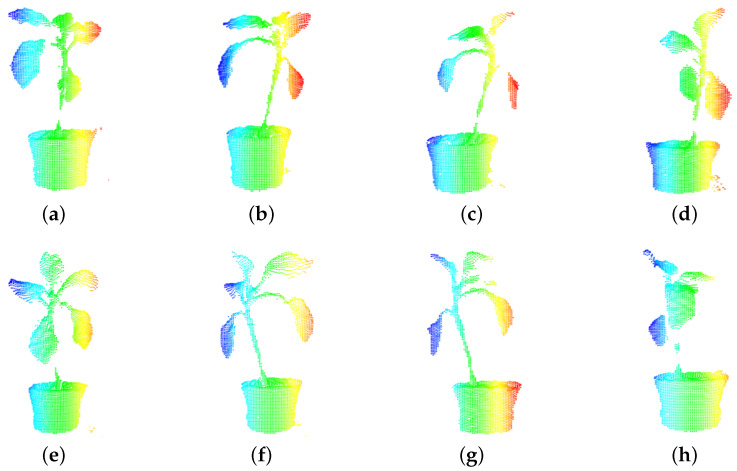
Color render plant’s point clouds of $angle_{0-7}$ according to *x*-axis direction. (**a**) point cloud of $angle_{0}$; (**b**) point cloud of $angle_{1}$; (**c**) point cloud of $angle_{2}$; (**d**) point cloud of $angle_{3}$; (**e**) point cloud of $angle_{4}$; (**f**) point cloud of $angle_{5}$; (**g**) point cloud of $angle_{6}$; (**h**) point cloud of $angle_{7}$.

Place the plant on the turntable and record the initial point cloud of the plant. Turn the turntable clockwise 45°, then stop the turntable and record the data as a piece of point cloud. Stop running until the turntable rotates 360° and 8 piece of point clouds are obtained. The original point cloud contains invalid data, so in this experiment the outlier points should be removed by statistical outlier filter [44], the distance $d_{i}$ from the query point to all the neighboring points is calculated, and the threshold standard deviation ε is set to judge whether the point is an outlier. Eight pieces of point clouds are registered by iterative closest point (ICP) [45] algorithm to obtain the initial three-dimensional point cloud model. In Figure 3, eight pieces of point clouds are obtained from different directions, related to the rotation angle of the turntable, $angle_{0}$ means the turntable rotates 0°, $angle_{1}$ means rotating 45°, *angle*_2_ means rotating 90°, *angle*_3_ means rotating 135°, *angle*_4_ means rotating 180°, *angle*_5_ means rotating 225°, *angle*_6_ means rotating 270°, *angle*_7_ means rotating 315°, *angle*_n_ is recorded every 45° (n ∈ [0, 7]) [46]. The point cloud of the turntable and the surrounding scene is marked as $p_{b}$, the point cloud of ROI from each angle can be obtained by subtracting $p_{b}$. Figure 3 shows the plant’s point clouds obtained after rotating the turntable 8 times interval, and the three-dimensional points of ROI are color-rendered according to *x*-axis for displaying clearly. The reason for selecting eight directions to collect experimental data is that the two adjacent point clouds can fill the holes at the edge of the point cloud after registering, so the three-dimensional point cloud can reduce the influence of holes on accuracy.

Plant reconstruction is divided into two parts in this research. The first part is to register *angle*_0_ with 7, 1, 2 and *angle*_4_ with 3, 5, 6 separately. The former result is called $Pos_{0}$ and the latter result is called $Pos_{1}$. The second part is to stitch the $Pos_{0}$ and $Pos_{1}$ point clouds to get a complete 360° reconstruction model. Here, the commonly used stitching method is the ICP algorithm, and this method has many advantages. Not only the registration between point set is considered, but also the registration from point set to model and model to model is also considered in the ICP algorithm.

The basic principle of the ICP algorithm is—in the target point cloud $P_{\left(i\right)}$ and the source point cloud $Q_{\left(i\right)}$, find the nearest neighbor point $\left(p_{i},q_{i}\right)$ according to certain constraints, and then calculate the optimal matching parameters *R* and *t* to make the error function minimum. The error function is E(R,t) in Equation (1) [45],

(1) $$E\left(R,t\right)=\frac{1}{n}\sum_{i=1}^{n}{∥q_{i}-\left(Rp_{i}+t\right)∥}^{2}$$

where *n* is the number of nearest neighbor pairs, $p_{i}$ is the point in the target point cloud *P*, $q_{i}$ is the nearest point of the source point cloud *Q*, *R* is the rotation matrix, *t* is the translation vector. The traditional ICP algorithm can be summarized in two steps—(1) compute correspondences between the two point clouds. (2) calculate the transformation that minimizes the distance between the corresponding points. The disadvantages [47] of this method are as follows—noise or abnormal data may cause the algorithm to fail to converge; the selection of the initial value has an important impact on the final registration results. The algorithm focuses on the similarity between the target and the source, and takes the minimum error as the evaluation standard, ignores whether there is a location relationship between the target point cloud and the source point cloud.

The improved ICP algorithm in this paper is more suitable for registering point clouds from different directions using a turntable. This stitching method first uses a rotation matrix and a translation vector to process the relationship between adjacent point clouds, then uses the ICP algorithm to iteratively obtain *R*, *t*. Obviously, the point clouds in eight directions obtained in this paper have positional relations. The point clouds in our experiment are formed by multiple rotations from the same plant, plant leaves from different directions are similar in shape, which can cause the ICP algorithm falling into local optimality, so this algorithm is improved. According to the position relationship of adjacent patch point clouds, firstly, rotate the source point cloud at angle $\theta_{1}$, then perform ICP registration of each point cloud, the following formula is in Equation (2),

(2) $$\begin{array}{cc} \; & \omega_{1}=\left[\begin{array}{ccc} cos\theta_{1} & 0 & sin\theta_{1}\\ 0 & 1 & 0\\ -sin\theta_{1} & 0 & cos\theta_{1} \end{array}\right],\sigma_{1}=\left(x_{t}-x_{s},y_{t}-y_{s},z_{t}-z_{s}\right)\\ \; & E\left(R_{1},t_{1}\right)=\frac{1}{n}\sum_{i=1}^{n}{∥q_{i}-\left(R_{1}\left(\omega_{1}p_{i}+\sigma_{1}\right)+t_{1}\right)∥}^{2} \end{array}$$

where $\left(x_{t},y_{t},z_{t}\right)$ is the centroid of the target point cloud, $\left(x_{s},y_{s},z_{s}\right)$ is the centroid of the source point cloud, $\omega_{1}$ is the rotation matrix around the *y*-axis for angle $\theta_{1}$, and the value of $\omega_{1}$ is based on the rotation relationship of target point cloud and source point cloud. After obtaining the centroid point three-dimensional vector of 8 pieces of point clouds, calculate the distance $\sigma_{1}$ between target point cloud and source point cloud, take target point cloud as the registration center, move source point cloud to the centriod coordinate point according to the distance $\sigma_{1}$.

**Figure 4:**
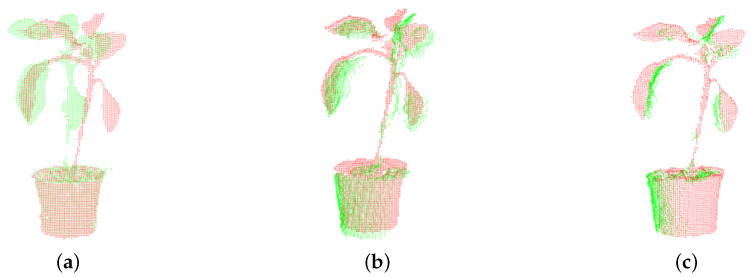
Comparison registering point cloud of $angle_{0}$ and $angle_{1}$ by traditional ICP algorithm with our algorithm. (**a**) Point cloud of $angle_{0}$ and $angle_{1}$. (**b**) Registering point cloud of $angle_{0}$ and $angle_{1}$ by traditional algorithm. (**c**) Registering point cloud of $angle_{0}$ and $angle_{1}$ by our algorithm.

In this experiment, take $angle_{0}$ as the target point, rotate $angle_{7,1,2}$ to register the two point clouds respectively; take $angle_{4}$ as the target point, rotate $angle_{3,5,6}$ to register the two point clouds respectively. For example, take $angle_{1}$ as the source point cloud, and $angle_{0}$ as the target point cloud in Figure 4a, color green represents $angle_{0}$, red represents $angle_{1}$, the relationship between them is rotating $angle_{1}$ of 45° anticlockwise along the $angle_{0}$, so $\theta_{1}$ is 45°. The iterations number of matching target and source point cloud *n* is 1000 times. The traditional ICP algorithm cannot judge the relationship between source point cloud and target point cloud as in Figure 4b, it shows that the stem and leaves do not match correctly and the position relationships of point clouds are neglected in the matching process, so the stitching error is large. The location relationship between the source and target point cloud should be determined in this research, that is, applying the ICP algorithm after rotation $\theta_{1}$, and the results are shown in Figure 4c by our method, after 1000 iterations, the leaves and stem match well. According to the rotation direction and angle of the turntable, the problem of ignoring the relationship between target point cloud and original point cloud in ICP registration can be solved by calculating the rotation angle in advance when registering the adjacent point clouds.

Finally, the result of the point cloud should be merged. The merged point cloud based on $angle_{0}$ is $Pos_{0}$, and the merged point cloud based on $angle_{4}$ is $Pos_{1}$. Point cloud $Pos_{0}$ are processed by using Equation (2), rotate $angle_{1}$ counterclockwise 45° and match with $angle_{0}$; rotate *angle*_2_ counterclockwise 90° and match with *angle*_0_; rotate *angle*_7_ clockwise 45° and match with label 0. Then, merge the results as $Pos_{0}$. Similarly, point cloud $Pos_{1}$ are processed in the same way, rotate label 5 counterclockwise 45° and match with label 4; rotate label 6 counterclockwise 90° and match with $angle_{4}$; rotate *angle*_3_ clockwise 45° and match with *angle*_4_. Finally, merge the results as *Pos*_1_.

Similarly, after obtaining *Pos*_0_ and $Pos_{1}$ point cloud, the ICP method cannot be used to match them directly, because $Pos_{0}$ and $Pos_{1}$ are in the different 3D coordinate systems, it should rotate $Pos_{1}$ by 180${}^{\circ }$ and keep it in the same coordinate system with $Pos_{0}$.

**Figure 5:**
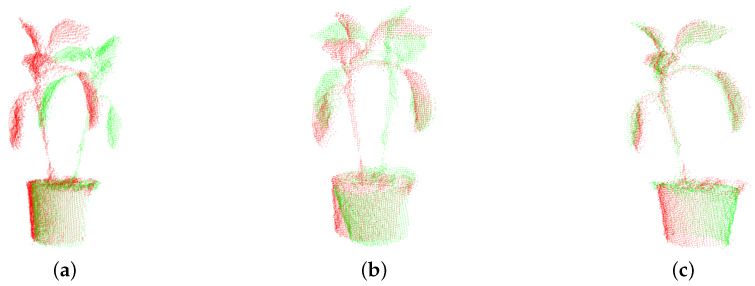
Comparison registering $Pos_{0}$ and $Pos_{1}$ point cloud by traditional ICP algorithm with our algorithm. (**a**) $Pos_{0}$ and $Pos_{1}$ point cloud. (**b**) Registering $Pos_{0}$ and $Pos_{1}$ point cloud by traditional ICP algorithm. (**c**) Registering $Pos_{0}$ and $Pos_{1}$ point cloud by our ICP algorithm.

For the $Pos_{0}$ and $Pos_{1}$ point clouds in Figure 5a, color red represents $Pos_{0}$, green represents $Pos_{1}$, half of the 360° model has been registered separately, so the correct registering of them directly affected the accuracy of the whole model. Because of the randomness in the growth of leaves, the centroids of $Pos_{0}$ and $Pos_{1}$ could not overlap. If ICP registration is directly used for registering, it will fall into local optimal and mismatching, as shown in the Figure 5b, which cannot reflect the relationship between $Pos_{0}$ and $Pos_{1}$ point cloud correctly. For this situation, our approach is to extract the difference between $Pos_{0}$ and $Pos_{1}$ centroid, move the target point cloud to the source point cloud, that is the local movement between these two centroid vectors and then using the ICP method to obtain a complete plant point cloud model as shown in Figure 5c. The algorithm cannot fall into the local optimum, and the registration effect of $Pos_{0}$ and $Pos_{1}$ is better. Here, $\sigma_{2}$ is the difference between $Pos_{0}$ and $Pos_{1}$’s centroid vector, as in Equation (3):

(3) $$\begin{array}{cc} \; & \omega_{2}=\left[\begin{array}{ccc} cos\theta_{2} & 0 & sin\theta_{2}\\ 0 & 1 & 0\\ -sin\theta_{2} & 0 & cos\theta_{2} \end{array}\right],\sigma_{2}=\left(x_{pos0}-x_{pos1},y_{pos0}-y_{pos1},z_{pos0}-z_{pos1}\right)\\ \; & E\left(R_{2},t_{2}\right)=\frac{1}{n}\sum_{i=1}^{n}{∥q_{i}-\left(R_{2}\left(\omega_{2}p_{i}+\sigma_{2}\right)+t_{2}\right)∥}^{2} \end{array}$$

where $\left(x_{pos0},y_{pos0},z_{pos0}\right)$ is the centroid of $Pos_{0}$, $\left(x_{pos1},y_{pos1},z_{pos1}\right)$ is the centroid of $Pos_{1}$, $\omega_{2}$ is the rotation matrix around the *y*-axis for angle $\theta_{2}$, and $\theta_{2}$ is 180°. After obtaining the centroid point three-dimensional vector of $Pos_{0}$ and $Pos_{1}$, calculate the distance $\sigma_{2}$ of them, take $Pos_{0}$ as the registration center, move $Pos_{1}$ to the centriod coordinate point with $Pos_{0}$ according to the distance $\sigma_{2}$. At this time, the registration and establishment of the 3D model is completed.

**Figure 6:**
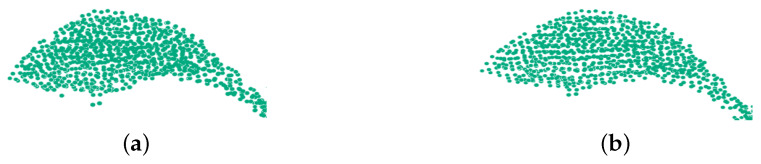
Comparison the original point cloud and moving least squares (MLS) processed point cloud. (**a**) Picking a leaf’s point cloud from the whole point cloud. (**b**) Smoothing these point clouds with MLS method.

To create complete models, glossy surfaces and occlusions in the point cloud must be accounted for. A solution is to use a resampling algorithm, which attempts to recreate the missing parts of the surface through high-order polynomial interpolation between surrounding data points. By performing the resampling process, small errors can be corrected, and the double-wall artifacts generated by registering 8 angles point cloud data together can be smoothed. Moving least squares (MLS) surface reconstruction [48] method can estimate normal vector based on polynomial reconstruction, and can also smooth and resample noisy data. In order to achieve the smoothness of the surface, the *K* nearest-neighbor radius [49] of the fitting is set as 0.5 mm, and the whole 360° point cloud is smoothed. As shown in Figure 6, a leaf is extracted from the whole point cloud registering by $Pos_{0}$ and $Pos_{1}$ point cloud, and the smooth point cloud obtained by MLS processing on it. MLS can smooth the surface and filter noise points.

Through the above steps, a whole point cloud model with less data, accurate normal and curvature variance is obtained, which is beneficial to the following operations such as feature point extraction and feature boundary line collection.

## 3. Measurement the Phenotypic Parameters of a Plant

### 3.1. Extraction of Boundary Points on a Plant Leaf

In this section, we discuss how to segment leaves and stems on a complete plant model, extract the contour of each leaf, and calculate the phenotype of the leaf according to the three-dimensional points of the contour. In this paper, kdtree is used to organize the data of leaf point cloud and realize fast nearest neighbor retrieval based on fast library for approximate nearest neighbors (FLANN) [50]. The spatial topological relationship between data points is established to facilitate *k*-nearest neighbor search.

The pepper plants are used as the research objects to generate three-dimensional models by eight pieces of point cloud. On the basis of the 3D model, the stem and leaves should be divided into different parts, so the phenotypic characteristics of each leaf can be calculated. The leaves for measurement are extracted from pepper plants by the locally convex connected patches method (LCCP) [51]. The principle of LCCP method is, for the plant model, firstly to calculate the convexity-concavity relationship of adjacent patches. The relationship is judged by extended connectivity criterion and sanity criterion. Extended connectivity criterion uses the angle between the center line vector $\vec{x_{1}}$, $\vec{x_{2}}$ and the normal vector $\vec{n_{1}}$, $\vec{n_{2}}$ of the adjacent patches. The vector from $\vec{n_{1}}$ to $\vec{n_{2}}$ is set to $\vec{t}$, the angle between $\vec{t}$ and $\vec{n_{1}}$ is $a_{1}$, and the angle between $\vec{t}$ and $\vec{n_{2}}$ is $a_{2}$. Obviously, if $a_{1}$ > $a_{2}$, the relationship of the two patches $\vec{p_{i}}$, $\vec{p_{j}}$ is concave, otherwise is convex. $\vec{d}$ is the difference between the vector $\vec{x_{1}}$, $\vec{x_{2}}$, $\vec{s}$ is the cross product between the vector $\vec{n_{1}}$, $\vec{n_{2}}$. If the relationship of the two patches is convex, it should be further judged by sanity criterion. When the angle between $\vec{d}$ and $\vec{s}$ is greater than the threshold δ, it can be sure that the relationship between them is convex, otherwise is concave.

**Figure 7:**
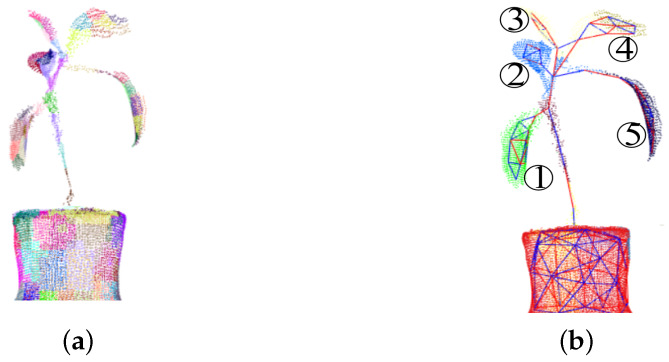
Segementation leaves and stem with locally convex connected patches method (LCCP) algorithm. (**a**) Segementation plant’s point cloud into small regions. (**b**) Small regions into larger objects by convexity-concavity relationship.

After marking the concavity-convexity relationship of each adjacent small region, the region growth algorithm is used to cluster the small regions in Figure 7a into larger objects in Figure 7b, representing by small blocks of different colors. This algorithm restricted by the convexity of small regions can clearly distinguish the boundary of stem and leaves as shown in Figure 7b, that red line represents concavity and blue line represents convexity between adjacent patches. There is obvious concavity and convexity change at the junction of fruit stem and leaves, which meets the criteria of LCCP segmentation.

The convexity-concavity relationship of the plant point cloud is obtained by LCCP algorithm, which displays in different colors. From the effect of segmentation, the closer the leaves are to the bottom of the plant, as shown in Figure 7b, the relationship between leaves and stem are more obvious; while the closer the leaves are to the top of the plant, for example, the relationship between leaf 3 and leaf 4 is not obvious, so they could not be segmented correctly by the LCCP method. Because the leaves at the top of the plant are closer towards the stem, they are treated as a whole, so it is necessary to manually segment the leaves.

**Figure 8:**
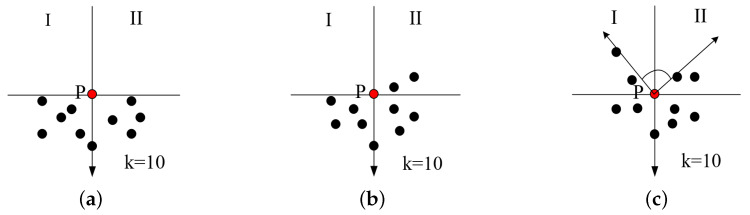
Three judgment conditions for obtaining the boundary points from the three-dimensional point cloud. (**a**) both areas have no points; (**b**) either one of the areas has no points; (**c**) the angle is smaller than ϵ.

For each divided leaf, the boundary condition [52] is used to determine whether the three-dimensional point of the leaf is an internal point or external contour point. For any point *p*, a tangent plane is established according to the point and its *k* neighborhood points, and project each point on this plane; then, judge whether the point *p* is a boundary feature point, repeat the above operations until all points are judged, and the boundary feature point set is obtained. When the target point is a boundary point, only a few or no points will appear in its upper region. According to this phenomenon, the discrimination mechanism of boundary feature points can be established. As shown in Figure 8, the upper part of point *P* is divided into two areas I and II by the vertical line and the horizontal line passing through point *p*. *p* is a boundary feature point can be distinguished according to whether there are data points in these two areas. Specifically, according to the boundary determination method, the edge point is the key to get the phenotype of leaf, taking k=10 as an example, it can be divided into three situations: (1) there are no points in the either areas, *p* is the boundary point; (2) there are no points in one area, *p* is the boundary point; (3) there are points in both areas, find out the vector closest to the vertical line of these two areas, and the angle between them. If it is greater than the set threshold ϵ, *p* is the boundary point, threshold is $\frac{\pi }{2}$ in this paper. In all three cases, the points are boundary points and need to be preserved. According to this method, edge extraction is performed on the segmented 3D leaves to extract points.

**Figure 9:**
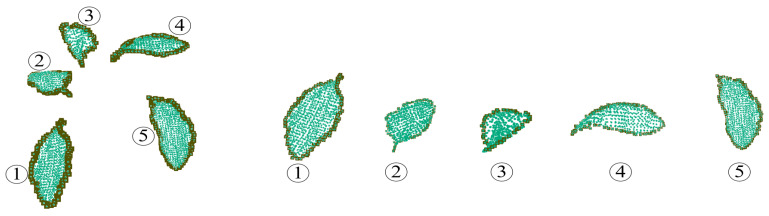
Extraction edge points of leaves by boundary conditions method.

The boundary points of the divided leaf are the basis of calculating the length and width of each leaf. Taking the green pepper plant at seeding stage as the research object, the LCCP algorithm is used to segment the leaves and stem, the boundary point judgment method is used to get the internal point and the external point, and then the edge points of each leaf are obtained, the leaves and stem are displayed in different colors. There are five leaves, and the edge points are displayed in black in Figure 9, which are the key points to obtain the leaf’s phenotypic parameters.

### 3.2. Calculation the Length, Width and Surface Area of Leaf

**Figure 10:**
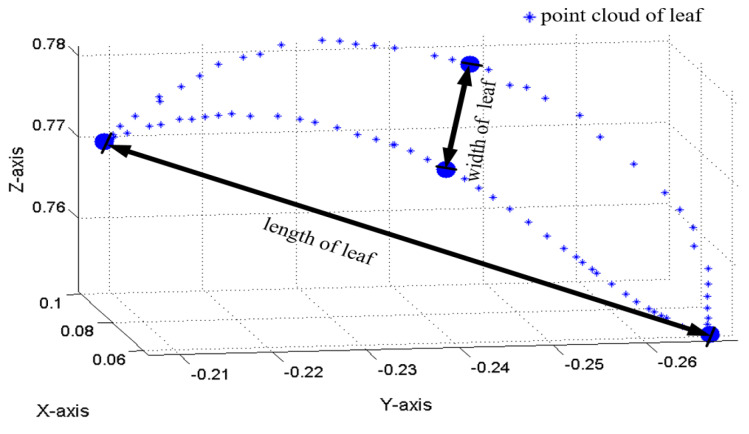
Fitting length and width of leaf with Euclidean distance algorithm.

Calculate the length and width according to the contour of each leaf. On the three-dimensional points of the boundary, manually select $p_{top}$ at the top of the tip and $p_{bottom}$ at the bottom of the leaf to calculate the Euclidean distance between them as the length of the leaf; and select the maximum distance perpendicular to leaf length on the edge point as $p_{side1}$ and $p_{side2}$ respectively to calculate the Euclidean distance between them as the width of the leaf in Equation (4),

(4) $$\begin{array}{c} l_{leaf}=\sqrt{{\left(x_{t}-x_{b}\right)}^{2}+{\left(y_{t}-y_{b}\right)}^{2}+{\left(z_{t}-z_{b}\right)}^{2}}\;\\ w_{leaf}=\sqrt{{\left(x_{1}-x_{2}\right)}^{2}+{\left(x_{1}-x_{2}\right)}^{2}+{\left(x_{1}-x_{2}\right)}^{2}}, \end{array}$$

where $\left(x_{t},y_{t},z_{t}\right)$ is the coordinate of $p_{top}$, $\left(x_{b},y_{b},z_{b}\right)$ is the coordinate of $p_{bottom}$, $\left(x_{1},y_{1},z_{1}\right)$ is the coordinate of $p_{side1}$, and $\left(x_{2},y_{2},z_{2}\right)$ is the coordinate of $p_{side2}$. $l_{leaf}$ is the length of leaf, $w_{leaf}$ is the width of leaf. In Figure 10, the blue three-dimensional points are the leaf contour obtained by boundary conditions, it is shown that the length and width of leaf can be obtained by using the Euclidean distance method, where length refers to the three-dimensional distance between the top and the bottom of the leaf, and width refers to the three-dimensional distance between the midpoint on both sides of the leaf.

In the 3D plant model, (x,y) of the point cloud coordinates (x,y,z) can be regarded as a coordinate pair obtained by sampling the coordinates on the xOy plane. For grid sample points, it should generate two matrices of the same size with the vector *x* as the rows and the vector *y* as the columns, where the rows of *x* start from the minimum value $x_{min}$ to the maximum value $x_{max}$ of the model, and generate data every 0.1 mm, which integrate as Matrix XI; Similarly, the columns of y start from the minimum value $y_{min}$ to the maximum value $y_{max}$, and generate data every 0.1 mm, which integrate as matrix YI. XI and YI form a uniform grid. XI is a row vector that determines a matrix with a fixed number of columns, YI is a column vector that determines a matrix with a fixed number of rows. Fit the surface (XI,YI,ZI) formed by z=f(x,y) to the scattered data in the vector (x,y,z). Perform cubic interpolation of data on the surface at the query point specified by (XI,YI) and return the interpolated value ZI to generate a smooth surface. The surface passes through the data points defined by (x,y) to form a complete mesh surface.

The leaf area is calculated according to the three-dimensional points. For the reconstructed leaf surface, the area is calculated by surface integral algorithm, and the area of the small rectangular block is added to get the surface area. When calculating the gradient, the step size needs to correspond to the small rectangular block’s length $d_{x}$ and width $d_{y}$. $d_{x}$ and $d_{y}$ in the Equation (5) are the slopes of rectangular blocks in *x*-axis and *y*-axis direction respectively. The area obtained from the integration of surface *s* on region $D_{xy}$ is:

(5) $$A={\;\int \int \;}_{D_{xy}}\sqrt{1+{\left(\frac{\partial z}{\partial x}\right)}^{2}+{\left(\frac{\partial z}{\partial y}\right)}^{2}}\;dx\;dy.$$

**Figure 11:**
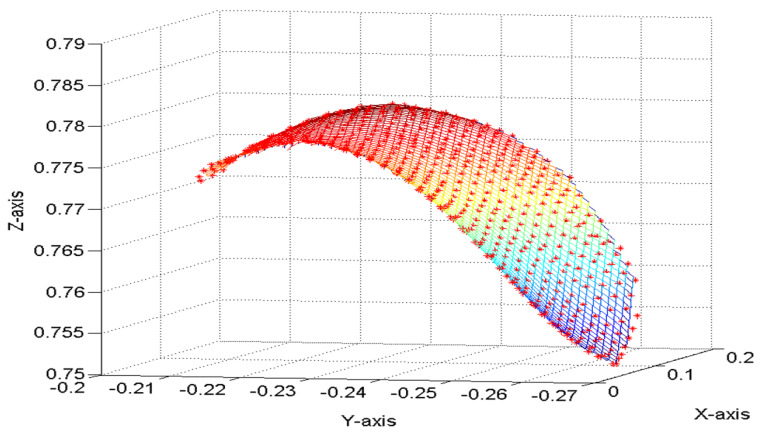
Fitting surface of leaf with double integral algorithm.

The fitted surface of leaf is as shown in Figure 11, the leaf is a closed surface, and the value of $d_{x}$ and $d_{y}$ are set at 0.0001, that means, the area of small square shall be obtained in every step of 0.1 mm.

### 3.3. Measurement the Height of a Plant

The height of the plant is obtained by vertical distance along *y* direction, by comparing all points’ three-dimensional coordinates, the maximum value $y_{i_{max}}$ and minimum value $y_{i_{min}}$ value along the *y*-directions are calculated in Equation (6). The height of the plant *H* is presented with flowerpot, $H_{1}$ is the vertical distance of the whole point cloud. Because the height of the flowerpot $H_{2}$ is included in the height $H_{1}$, in order to get the true height of the plant, the actual height of the flowerpot should be subtracted here. In Equation (7), the height of the cuboid $H_{1}$ is the sum of the plant *H* and the flowerpot $H_{2}$, and the height of the flowerpot has been subtracted in this experiment.

(6) $$\begin{array}{cc} \; & \;\;\;y_{i_{min}}\leq y\leq y_{i_{max},\;}\left(1\leq i\leq n\right)\\ \; & \;\;\;H_{1}=y_{max}-y_{min}, \end{array}$$

(7) $$\begin{array}{cc} \; & H_{2}=y_{maxpot}-y_{minpot}\\ \; & H=H_{1}-H_{2}\;\;. \end{array}$$

The height of the flowerpot $H_{2}$ can be obtained in Figure 7b after the result of LCCP segmentation. The concavity-convexity relationship between the flowerpot and the stem of the plant separate the two into different spaces. Therefore, the maximum value $y_{maxpot}$ and the minimum value $y_{minpot}$ of flowerpot in the *y*-axis direction can be used as the vertical distance of the flowerpot.

**Figure 12:**
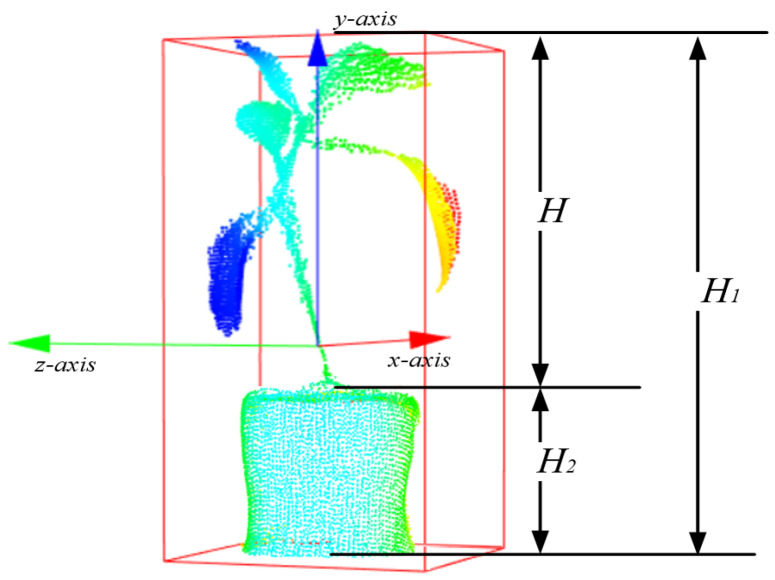
Calculation the plant’s height by vertical distance method based on *y*-axis.

In Figure 12, the red arrow represents the *x*-direction, the blue arrow represents the *y*-direction, and the green arrow represents the *z*-direction, the external minimum cuboid is drawn by the main direction coordinate system. Then, for the *y*-axis of interest in this paper, the actual height difference between the cuboid’s vertical distance and the flowerpot is obtained to get the height of the plant.

## 4. Experiment

The experimental results are analyzed by comparing the measured values with actual values. Plant height, leaf length, width and surface area are the main analysis indexes. According to the experiment in this paper, the absolute error (AE) of plant height, the mean absolute error (MAE), root mean square error (RMSE) of leaf length, leaf width and surface area of each plant are calculated. Ten seedling plants of pepper are selected as the test objects, denoted as $plant_{n}$, (n ∈ [1, 10]).

**Figure 13:**
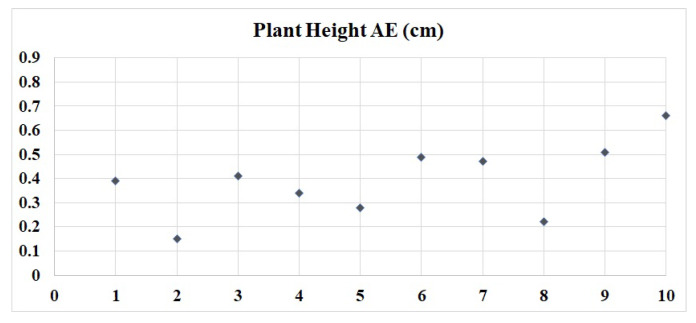
Values of absolute error (AE) for plant’s height.

Based on Kinect V2, the 3D model of green pepper was constructed and the plant’s phenotypic parameters including plant’s height, leaf’s length, width and area were measured. The AE of plant height is the difference between the actual value and the measured value, and the MAE of plant height is the average value of 10 plants’ AE. The minimum actual value of plant height of 10 green peppers is 11.12 cm, and the maximum actual value is 21.09 cm. In Figure 13, the maximum AE of plant height is 0.66 cm, the minimum AE is 0.15 cm, and the MAE of plant height is 0.392 cm.

**Figure 14:**
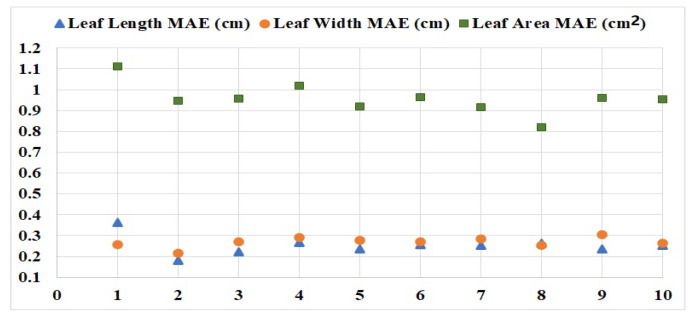
Values of mean absolute error (MAE) for leaves’ phenotypic characteristics.

The leaf phenotype is an important growth parameter for pepper seedling plants. In this paper, pepper plants’ point clouds were segmented by the LCCP method, four leaves of each green pepper were selected as a whole for measurement, and recorded these as measured values. The average length, width, area errors of four leaves for each green pepper are denoted by $MAE_{l}$, $MAE_{w}$ and $MAE_{a}$. The maximum actual value of $MAE_{l}$ is 0.365 cm, and the minimum actual value of $MAE_{l}$ is 0.1825 cm; the maximum actual value of $MAE_{w}$ is 0.305 cm, and the minimum actual value of $MAE_{w}$ is 0.2175 cm; the maximum actual value of $MAE_{a}$ is 1.1125 cm^2^, and the minimum actual value of $MAE_{a}$ is 0.821 cm^2^. The MAE of the leaf’s length, width and area of these 10 peppers are denoted as $MAE_{leaf^{\prime }s\;length}$, $MAE_{leaf^{\prime }s\;width}$ and $MAE_{leaf^{\prime }s\;area}$. In Figure 14, $MAE_{leaf^{\prime }s\;length}$ is 0.2537 cm, $MAE_{leaf^{\prime }s\;width}$ is 0.2676 cm and $MAE_{leaf^{\prime }s\;area}$ is 0.956 cm^2^. $MAE_{l}$ and $MAE_{w}$ are directly proportional to $MAE_{a}$, because the leaf’s area is related to the value of length and width, with the increase of $MAE_{l}$ and $MAE_{w}$, the $MAE_{a}$ become larger.

In Table 1 the measurement accuracy in this paper was compared with other references. In these methods, Reference [24,25] use binocular vision to obtain the point cloud, Reference [28] uses the 3D laser scanner to obtain the point cloud, and References [3,15] use Kinect to obtain point cloud of regions of interest (ROIs). In Reference [25], VisualSFM was used to register the point cloud of eggplant, pepper and cucumber, segmented these plants by region growing method, and measured the leave’s parameters of these plants. In Reference [24] 3DSOM was used to register the point cloud of Gossypium hirsutum, segmented the plant by region growing and mesh method, and measured the leaves’ parameters of the plant. In Reference [28], target ball extraction algorithm [44] was used to register the point cloud of apple tree branches, segmented the plant by LCCP and Kmeans clustering algorithm, and measure the leaves’ parameters of the plant. In Reference [3], higher solution RGB images was used to register the point cloud of pepper, measured the leaves’ parameters and plant’s height. In Reference [15] segmented the cucumber plants using the Euclidean clustering method, and using the vertical instance method to obtain the height of the plants. Compared with other methods, MAE and RMSE of our method have lower comprehensive errors, and our method has advantages of leaf’s surface area measurement. The measurement errors of plant height, leaf length and width are also at the same level with other measurement methods.

## 5. Conclusions

The research objects of this paper are the plants in the early growing period. Taking green pepper plants as an example, each leaf plays an important role in the growth of plants. The information of the sum of single leaf on the whole plant is useful for assessing the growth state. The established three-dimensional model is the key to study the whole plant information. Using depth camera and turntable to complete point cloud acquisition and registration is the basic operation for plant segmentation, height measurement and a leaf’s phenotypic features extraction. This paper processes the point clouds according to the characteristics of rotation by equal intervals, which can avoid the process of registration falling into the local optimal solution and ignoring the overall information. The plant model obtained by rotation and registration can reduce the inaccuracy of leaf shape description due to different shooting angles.

The use of the LCCP algorithm to segment the plant point cloud model is a popular three-dimensional point segmentation method. This method avoids the process of selecting initial points of region growing algorithm. The LCCP algorithm uses concave-convex features to segment points, while there is a natural convexity-concavity relationship between leaves and stems. This is another reason why we choose this segmentation algorithm to segment the plant’s point cloud but the convexity-concavity relationship of the adjacent leaves’ point clouds is not obvious, sometimes the young leaves on the top of the plant need to be selected manually. For plants in the growing period, the Leaf Area Index has a greater influence on the growth state of the plant. At this time, the leaves of the plant are dense and the shape of each leaf is larger. Obtaining each leaf individually is impossible, because there is often occlusion between dense leaves, which cannot be reduced by changing the shooting angle. Therefore, it is necessary to find new phenotypic features that can represent plant growth instead of leaves’ parameters.

Our experiment was done indoors, and the shooting camera and the object were placed horizontally, so the three-dimensional points of *y*-axis coordinate system was used to determine the plant height. At present we have not reproduced the experiment outdoors. Because the outdoor scene is complicated as the plants are generally in the ground, it is difficult to complete the 3D modeling of plants according to the proposed rotation method. However, this paper proposed a general method, which can be extended to the related fields of 3D model building indoors.

## Figures and Tables

**Table 1:** MAE, RMSE between this paper and other papers in the terms of plant height, leaf length, leaf width and leaf area.

Expriment	Object	Index	Length (cm)	Width (cm)	Area (cm^2^)	Plant Height (cm)
This Paper	Pepper	RMSE	0.2639	0.2735	0.964	0.417
Fang [25]	Cucumber		0.28	0.32	4.34	-
	Eggplant		0.16	0.23	3.89	-
	Pepper		0.33	0.15	1.33	-
Paproki [24]	Gossypium hirsutum		0.97	0.728	-	1.9
Liu [28]	apple tree		0.59	0.538	-	-
This Paper	Pepper	MAE	0.2537	0.2676	0.957	0.392
Kaiyan [3]	Pepper		0.183	0.124	-	0.344
Yang [15]	Cucumber		-	-	-	0.23
Liu [28]	apple tree		0.55	0.51	-	-

## Abbreviations

The following abbreviations are used in this manuscript:

3D	Three-dimensional
RGB-D	Red, Green, Blue, and Depth
TOF	Time of Flight
ROI	Region of Interest
v2.0	Version 2.0
ICP	Iterative Closet Point
MLS	Moving Least Squares
Pos	Position
FLANN	Fast Library for Approximate Nearest Neighbors
LCCP	Locally Convex Connected Patches
min	minimum
max	maximum
AE	Absolute Error
RMSE	Root Mean Squared Error
MAE	Mean Absolute Error
